# Humoral Response to the Third Dose of SARS-CoV-2 Vaccine Among Dialysis Patients: A Breakthrough Infection Case–Control Study

**DOI:** 10.3390/vaccines13090935

**Published:** 2025-09-01

**Authors:** Francesca Colavita, Concetta Castilletti, Giulia Matusali, Silvia Accordini, Salvatore De Masi, Roberto Da Cas, Natasha Gianesini, Giovanni Baglio, Massimo Francalancia, Giuseppe Traversa, Flavia Chiarotti, Silvia Meschi, Elvira Bianco, Mario Salomone, Alfonso Mele, Piergiorgio Messa, Carmine Zoccali, Francesca Menniti Ippolito, the COVIDVaxDia Study Group

**Affiliations:** 1 Laboratory of Virology, National Institute for Infectious Diseases Lazzaro Spallanzani, IRCCS, 00149 Rome, Italy; francesca.colavita@inmi.it (F.C.); giulia.matusali@inmi.it (G.M.); massimo.francalancia@ifo.it (M.F.); silvia.meschi@inmi.it (S.M.); 2 Department of Infectious, Tropical Diseases and Microbiology, IRCCS Sacro Cuore Don Calabria Hospital, Negrar di Valpolicella, 37024 Verona, Italy; concetta.castilletti@sacrocuore.it (C.C.); silvia.accordini@sacrocuore.it (S.A.); natasha.gianesini@sacrocuore.it (N.G.); 3 Clinical Trial Centre, Careggi Hospital, 50134 Florence, Italy; demasis@aou-careggi.toscana.it; 4 Italian National Institute of Health, 00161 Rome, Italy; giuseppetraversa24@gmail.com (G.T.); flavia.chiarotti.lavoro@gmail.com (F.C.); alfonso.mele2013@gmail.com (A.M.); francesca.menniti@iss.it (F.M.I.); 5 Italian National Agency for Regional Healthcare Services, 00187 Rome, Italy; baglio@agenas.it; 6 Careggi Hospital, 50134 Florence, Italy; bianco@aou-careggi.toscana.it; 7 Unit of Nephrology and Dialysis, Chieri and Moncalieri Hospitals, 10024 Turin, Italy; mario.salomone54@gmail.com; 8 Unit of Nephrology, Dialysis, and Renal Transplant, Fondazione IRCCS Ca’ Granda Ospedale Maggiore Policlinico, 20162 Milan, Italy; piergiorgio.messa@gmail.com; 9 Renal Research Institute, New York, NY 10065, USA; carmine.zoccali@icloud.com; 10 Istituto di Biologia e Genetica Molecolare (BIOGEM), 83031 Ariano Irpino, Italy; 11 Associazione Ipertensione, Nefrologia e Trapianto Renale (IPNET) c/o Nefrologia, Grande Ospedale Metropolitano, 89133 Reggio Calabria, Italy

**Keywords:** SARS-CoV-2 vaccine, COVID-19, dialysis, humoral response, neutralizing antibody

## Abstract

**Background:** COVID-19 vaccination and subsequent booster doses became critical components of public health strategies to control the pandemic and reduce disease severity, especially in fragile individuals. Among these, subjects undergoing dialysis represent one of the highly vulnerable populations. **Methods:** We conducted a multicenter case–control study among dialysis patients between March 2021 and May 2022 (study population n = 3264). We evaluated anti-S/RBD-IgG and anti-SARS-CoV-2 neutralizing antibodies before (T3) and after (T4) the third dose in individuals with a COVID-19 diagnosis after the third dose (cases) and in those who did not report infection (controls). **Results:** The study included 187 cases and 150 controls. Serological analysis showed a significant increase (*p* < 0.001) in anti-SARS-CoV-2 antibody levels after the third vaccine dose (from T3 to T4) in both groups. At T3, with the same number of days between the second dose and T3, the antibody levels detected were significantly lower in cases as compared to controls. At T4, we observed similar antibody titers in the two groups. Notably, the mean difference in time from the third dose to T4 was significantly greater in controls (73.0 days vs. 36.7, *p* < 0.001), suggesting a reduced antibody waning in controls. Accordingly, multivariate analysis showed that the risk of infection was considerably reduced by the pre-third-dose antibody levels. **Conclusions:** This study reinforces the critical role of the humoral response in preventing infections in the vulnerable population of dialysis patients. Regular monitoring of antibody levels and timely administration of booster doses are essential to optimize protection in this group.

## 1. Introduction

The SARS-CoV-2 epidemic necessitates a comprehensive understanding of the immune responses triggered by vaccination, particularly in vulnerable populations. Patients undergoing dialysis for End-Stage Renal Disease (ESKD) due to Chronic Kidney Disease (CKD) stage G5D have faced severe challenges during the COVID-19 pandemic, with a reported 20% early mortality attributable to the virus, as noted in the European Registry of Dialysis and Transplantation [1]. This high mortality rate is attributed to increased exposure in healthcare settings, a high prevalence of comorbidities, and compromised immune function inherent to kidney failure [2,3]. As the pandemic evolved, vaccination and subsequent booster doses became critical components of public health strategies.

Although the definitive correlation of protection against infection has not yet been established, antibody levels—particularly neutralizing antibodies—are considered surrogate markers of protection immunity and are therefore widely used for post-vaccination monitoring. Previous studies showed that the antibody response is notably delayed in dialysis patients as compared to healthy individuals [4]. While COVID-19 vaccines have not significantly reduced infection rates, they have markedly decreased severe outcomes in this population. The antibody response rate in hemodialysis patients was reported to be 45% after the first dose and 89% after the second dose [2]. Studies assessing the efficacy of a booster dose in dialysis patients have demonstrated an increase in antibody levels [5,6,7,8,9], with a more pronounced response in those with initially low antibody levels or longer intervals between doses [10,11]. The booster dose has shown efficacy in seroconverting patients with previously low or absent responses [12], with similar outcomes observed in both hemodialysis and peritoneal dialysis patients [10,13]. Factors such as immunosuppressive therapy, obesity, advanced age, and the absence of prior SARS-CoV-2 infection or breakthrough infections can predict suboptimal immune responses [6,14,15]. In those with impaired renal function, uremia further hinders dendritic cell function, impacting vaccine efficacy [3,16].

Despite improvements in vaccination and emerging treatments, dialysis patients remain highly vulnerable to COVID-19, with ongoing uncertainty regarding optimal protective antibody titers. Specific SARS-CoV-2 neutralizing antibodies and anti-spike antibodies are identified by both the Federal Drug Administration (FDA) and European Medicines Agency (EMA) as surrogate markers and valuable correlates of protection [17]. Here, we report the data obtained from a national study conducted by the Italian National Institute of Health in collaboration with the Italian Society of Nephrology to contribute to the knowledge on prevention strategies during the COVD-19 pandemic in a fragile population. Although it is theoretically more correct to distinguish between “additional primary dose” as any extra dose administered to frail individuals in addition to the vaccination schedule and “additional dose” for immunocompetent subjects, in this study, the term booster will be used for both categories, as indicated by the Italian Ministry of Health [18]. This study focused on the vaccine humoral response and its association with breakthrough infections post-third vaccine dose.

## 2. Materials and Methods

### 2.1. Study Population

We conducted a multicenter study among dialysis patients, between 1 March 2021 and 31 May 2022, for the purpose of evaluating (i) vaccine immunogenicity by measuring the levels of specific SARS-CoV-2 neutralizing antibodies and anti-Spike/RBD IgG and (ii) the clinical efficacy and safety of COVID-19 vaccination. We invited all Italian dialysis centers to participate. We included individuals vaccinated against COVID-19 according to the schedule recommended by the Ministry of Health at the start of the study [19]. Patients were eligible to be included in the study if they were older than 16 years, on dialysis treatment for at least 3 months, and willing to undergo the required clinical and laboratory tests and provided informed consent. All patients who received at least one dose were asked to participate in the study. We excluded subjects with documented SARS-CoV-2 infection before the first dose or with a life expectancy ≤ 6 months (based on the clinical judgment of nephrologists at center level). We used an electronic Case Report Form (eCRF) for the collection of demographic and clinical information, vaccination status (including other vaccinations in addition to COVID-19), background diseases and ongoing therapies at the time of enrolment and after vaccination, hospitalizations, access to intensive care units, laboratory tests, adverse events, and study outcomes. The detection of SARS-CoV-2 infection and/or disease was recorded through routine surveillance activities in participating dialysis centers. Ascertainment of diagnosis was performed through routine antigenic/molecular tests or retrieved from clinical records for hospitalized patients. A subgroup of dialysis centers agreed to participate in a case–control study for the evaluation of the humoral immunological response by measuring the levels of SARS-CoV-2 specific anti-Spike/Receptor-Binding-Domain-IgG (anti-S/RBD-IgG) and SARS-CoV-2 neutralizing antibodies (nAb). For these individuals, we collected blood samples using a Clot Activator tube with Gel Separator, and serum was obtained by centrifuging for 30 min at 3500 rpm. Samples were aliquoted and stored at controlled temperature (−20 °C or −80 °C) until shipment to the Reference Laboratories for serological investigation.

### 2.2. Case–Control Study Design

Cases were people with a diagnosis of breakthrough COVID-19 after the third vaccine dose who were enrolled in the dialysis centers participating in the study [20]. Controls were people who did not have SARS-CoV-2 infection during the study period and had three vaccines doses like the cases. They were selected as a sample within the same clinical centers as the cases, with dates of vaccination and time of blood sample collection close to those of the cases. We could not identify the viral variants in each enrolled patient. However, since controls were selected among patients with a blood sample collection and vaccination date as close as possible to those of cases, and in the same geographical area, we are confident that the viral variants did not affect the comparisons between cases and controls. All enrolled patients were vaccinated with three doses of mRNA vaccines, recommended in Italy for this population. Levels of specific anti-SARS-CoV-2 antibodies, including anti-S/RBD-IgG, anti-Nucleocapsid-IgG (anti-N-IgG), and nAb, were evaluated before the third dose (T3) and at least 7 days after the third dose (T4). For the group of cases, T4 corresponded to the samples preceding the infection. Samples after T4 were not available in all cases and controls to perform further analyses. All subjects who resulted positive to the specific anti-N-IgG (positive: ≥1.4 S/C index) were excluded as suggestive of a previous SARS-CoV-2 infection.

### 2.3. SARS-CoV-2 Antibody Testing

We performed specific antibody assays using the same methods in two facilities: the Laboratory of Virology of the Lazzaro Spallanzani National Institute for Infectious Diseases IRCCS in Rome, and the Microbiology Laboratory of the IRCCS Sacro Cuore Don Calabria in Negrar di Valpolicella (VR). Methods used for antibody assays are described in Supplementary S1.

### 2.4. Sample Size

We calculated the size of the effect that the study would be able to capture as statistically significant in a Mann–Whitney U test (min ARE parent distribution) for the comparison of the average levels of antibodies in cases and controls, using Cohen’s d effect size formula with a two-tailed significance level equal to 0.05 and a power of 0.90. Cohen’s d is designed to estimate the effect size in the comparison of two groups. It takes the difference between two means and expresses it in standard deviation units. It tells how many standard deviations lie between the two means. We calculated Cohen’s d to test the hypothesis that a significant difference can be captured in a comparison of 150 cases and 150 controls; this resulted in an estimate of 0.404 (small–medium effect size). When comparing the antibody levels before and after the third dose, in 150 subjects (cases and controls separately), with the Wilcoxon matched pairs signed rank test with two-tailed significance level equal to 0.05 and a power of 0.90, the calculated value for Cohen’s d was equal to 0.287.

### 2.5. Statistical Analysis

Quantitative variables were summarized by means, standard deviations (SDs), medians, and ranges, while qualitative or categorized variables were summarized by absolute and percent frequencies. Anti-SARS-CoV-2 antibodies were calculated for each blood sample, in absolute values for IgG and as base-2 logarithms (log_2_) for SARS-CoV-2 nAb. The Mann–Whitney U non-parametric test was used to evaluate differences in quantitative variables between cases and controls. The Wilcoxon matched pairs signed rank test was used to compare the antibody levels in cases and in controls before and after the 3rd dose. Differences between cases and controls for qualitative or categorized variables were assessed by Fisher’s exact probability test. Finally, stepwise backward logistic regression (*p* remove = 0.100, *p* entry = 0.099) was used to assess the effect on the probability of being infected after the third vaccination (dependent variable) of Anti-S/RBD-IgG or log_2_ nAb levels (independent variables in separate models) measured pre- and post-third vaccination. We considered (a) sex, age, time from 2nd vaccination to T3, and time from 3rd vaccination to T4 (covariates forced in the model) and (b) heart disease, diabetes, arterial hypertension, autoimmune disease, neoplasia, and comorbidities (additional independent variables subjected to backward selection). Robust standard errors (SEs) of logistic regression coefficients were estimated clustering subjects by region, used as proxy for the level of circulating viruses. Odds ratios (ORs) with 95% confidence intervals (95%CIs) were reported. Statistical analyses were performed using STATA 16.1 (StataCorp LLC, College Station, TX, USA, rev. June 2023) and Prism 9.0 (GraphPad, La Jolla, CA, USA).

## 3. Results

**Figure 1:**
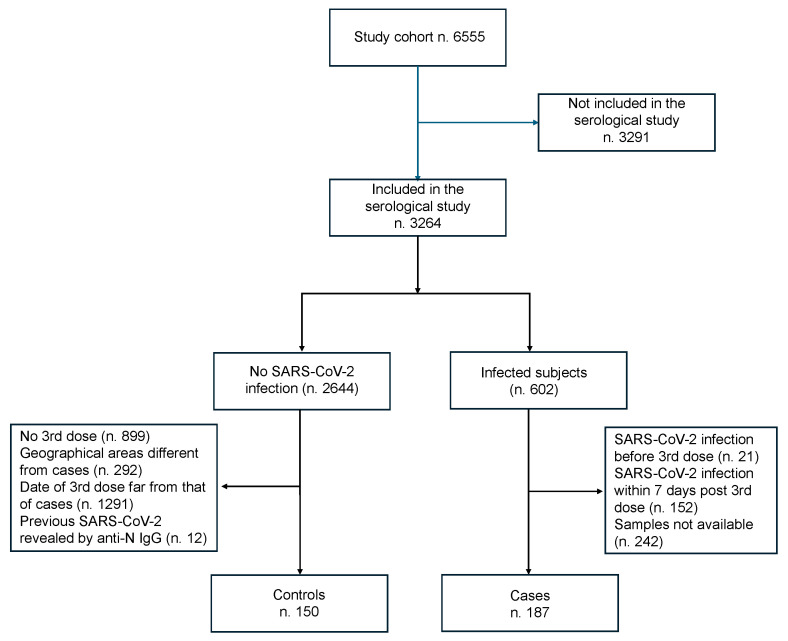
Flow-chart of the enrolment of the study cohort.

Out of 6555 enrolled patients included in the cohort study, 3264 subjects participated in the case–control serological study (602 SARS-CoV-2 infected and 2644 not infected). Included patients were enrolled in 67 clinical centers in 12 Italian regions, with a total of 15,997 blood samples collected at different time points. As shown in Figure 1, a total of 187 cases (infection from at least 7 days from the third dose) were selected (21 were excluded because of SARS-CoV-2 infection before the third dose, 152 because infection occurred within 7 days from the third dose of the vaccine, and 242 because their blood samples were not available). In total, 150 controls were selected (899 were excluded because they did not have the third dose; 292 were excluded due to being in geographical areas different from those of the cases; 1291 were excluded for having a third dose far from the corresponding date of cases; and 12 were excluded because a previous SARS-CoV-2 infection was revealed by anti-N IgG).

The laboratory at the IRCCS Sacro Cuore Don Calabria Hospital in Negrar di Valpolicella, Verona, analyzed 392 samples for anti-S/RBD-IgG and anti-N-IgG and 257 for nAb. The laboratory at the IRCCS INMI Lazzaro Spallanzani in Rome analyzed 580 samples for anti-S/RBD-IgG and anti-N-IgG and 515 for nAb. We describe the demographic and clinical characteristics of cases and controls in Table 1. Cases and controls were significantly different in terms of age, heart disease, diabetes, arterial hypertension, autoimmune diseases, neoplasia, and number of comorbidities. Autoimmune diseases, which can strongly affect the immune response to the vaccine, were used as a proxy of immunosuppressive treatments and were more frequent in the case group with respect to controls (9% vs. 3%, *p* = 0.021). The mean time (days) between the third vaccine dose and T4 was significantly higher in the control group with respect to cases (73.0 vs. 36.7, *p* < 0.001) (Table 1). In cases, the mean time between breakthrough infection and the third dose was 133 days (SD 46.4). Furthermore, all cases became infected after T4; the mean time (SD) between breakthrough infection and T4 was 99 days (46.5).

**Figure 2:**
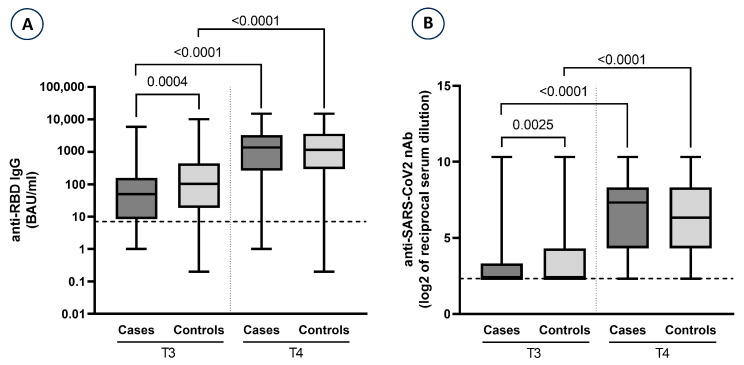
Distribution of anti-S/RBD-IgG (**A**) and log_2_ nAb (**B**) at T3 and T4 by cases and controls. nAb levels are presented as log_2_ scale. Graphs show median values, and error bars indicate range values. The dotted lines indicate the limit of antibody quantification. Mann–Whitney U non-parametric test was used to compare anti-RBD IgG and nAb levels in cases versus controls, while Wilcoxon matched-pairs signed rank test was used for comparison of the antibody titers before and after the 3rd dose. *p* < 0.05 was considered statistically significant.

The anti-S/RBD-IgG titer reflects the magnitude of the antibody response to the vaccine, and nAb titers measure functional immunity to SARS-CoV-2. We observed a statistically significant increase (*p* < 0.001) from T3 to T4 of anti-S/RBD-IgG and nAb levels in both groups (Figure 2). As shown in Table 2, the median level of anti-S/RBD-IgG was lower in cases with respect to controls at T3 (immediately preceding the administration of the third dose of vaccine) (49.6 vs. 102.1; *p* = 0.0004), while very similar at T4 (1363.9 vs. 1142.6; *p* = 0.9527). Also, nAb levels (expressed as log_2_) showed the same trend (Table 1).

In the multivariate logistic regression analysis, an increases of 100 units in the levels of anti-S/RBD-IgG pre- and post-third dose of vaccine was associated with a reduction in the risk of infection by about 3% (OR = 0.97, 95% CI 0.93–1.00) and 1% (OR = 0.99, 95% CI 0.93–1.00), respectively. Notably, in this type of analysis, the time from vaccination to sample collection (both second to T3 and third to T4) was equal. The risk of developing SARS-CoV-2 infection in diabetic patients was substantially increased (OR = 1.82, 95% CI: 1.10–3.02) and even more so in those with autoimmune diseases (OR = 3.02, 95% CI: 1.15–7.91). Neither neoplasia, the interval (days) between the second and the third dose, nor that after receiving the third dose was associated with the risk of developing COVID-19 (Table 3).

Similarly, the multivariate logistic regression analysis on log_2_ nAb levels showed that levels pre-third dose (T3) reduced the risk of infection by about 19% (OR = 0.81, 95% CI 0.73–0.90), and log_2_ nAb levels post the third dose of vaccine (T4) reduced the risk of infection by about 4% (OR = 0.96, 95% CI 0.83–1.11). The OR decreases by 19% or 4% (before and after the third dose, respectively, per unit increase in log_2_ nAb, corresponding to a doubling of nAb.

Moreover, patients with diabetes had almost double the risk of developing SARS-CoV-2 infection (OR = 1.97, 95% CI: 1.17–3.32), and patients with autoimmune diseases had almost three times the risk for the same outcome (OR = 2.90, 95% CI: 1.19–7.08) (Table 3).

## 4. Discussion

The current study provides an analysis of the humoral response to the third dose of the SARS-CoV-2 vaccine in a cohort of dialysis patients enrolled in Italy between 2021 and 2022. The antibody response was evaluated in individuals who did not report breakthrough COVID-19 after the third vaccine dose (control group) and in those who were infected with SARS-CoV-2 after the third dose (cases). Patients infected with SARS-CoV-2 prior to vaccination were excluded to avoid the confounding effect of hybrid immunity. Including these patients would have complicated the evaluation of the effect of the immune response solely driven by vaccination, thus compromising the validity of our case–control study design.

Our findings confirm a significant increase in antibody levels (*p* < 0.001) following the third COVID-19 vaccine dose both in controls and in cases, aligning with similar studies that demonstrated improved antibody responses in dialysis patients receiving booster vaccination [5,7]. The case–control evaluation showed that before the third dose (T3), with a similar mean time (days) from the second dose administration to the time of the blood draw, the control group showed a significantly higher titers of both anti-S/RBD-IgG antibodies (705.5 vs. 235.0, respectively, *p* = 0.0004) and nAb antibodies (3.79 vs. 3.02, respectively, *p* = 0.0025) as compared to cases. These findings suggest a better immunological status in controls after the second dose of COVID-19 vaccine and could explain the reason for the lack of SARS-CoV-2 infection in the later period [4,6,21,22]. The evaluation of the antibody levels after third dose administration showed similar titers for both anti-S/RBD-IgG (2454.8 in controls vs. 2291.7 in cases, *p* = 0.9527) and nAb (6.27 in controls and 6.23 in cases, respectively, *p* = 0.9002) between the two groups. Notably, the mean difference in time (days) between the third dose and T4 (for cases, the sample preceding the infection) was significantly greater in controls than in cases (73.0 in controls vs. 36.7 in cases, *p* < 0.001). The different time intervals between third dose and blood sample collection in cases and controls was considered acceptable for feasibility reasons. This could represent a possible bias. However, notwithstanding the difference observed between the mean time in cases and controls (36.7 vs. 73.0 days), the level of antibodies was higher in control patients, strengthening the potential protective role of antibodies regarding the occurrence of SARS-CoV-2 infection. Thus, this might indicate a longer time of antibody titer waning in controls than in cases.

Multivariate logistic regression analysis showed that nAb titers pre-third dose (T3) were associated with a reduction in the risk of infection by 19% (OR = 0.81, 95% CI 0.73–0.90) and post-third dose (T4) by 4%, (OR = 0.96, 95% CI 0.83–1.11) on log_2_ scale. Data on baseline immune parameters (e.g., lymphocyte counts, inflammatory markers) and T-cell response are lacking, thus limiting the ability to conduct a comprehensive analysis, as these factors may offer additional insights into post-vaccine immune responses that are not fully captured by antibody titers alone. Although adaptive immunity also plays an important role in vaccine-induced immunity, its assessment mainly focuses on humoral immunity. Extensive research studies have been conducted on SARS-CoV-2-related humoral and adaptive immunity, indicating their role in protecting against infection as well as the vaccine’s role in their induction. It is known that neutralizing antibody titers induced by both natural infection and vaccination decrease over time, increasing susceptibility to reinfection. In addition, SARS-CoV-2 undergoes rapid mutations that allow the virus to escape the antibody response. Both SARS-CoV-2-specific memory B cells and T cells respond rapidly to reinfections caused even by new variants, playing a key role in viral clearance, prevention of hospitalization and severe disease, and recognition of emerging variants [23].

Despite the overall positive response to vaccination, certain subgroups remain at higher risk for breakthrough infections. In this paper, it was not possible to analyze different types of dialysis (hemodiafiltration, peritoneal, etc.), transplant, or early-stage CKD cases separately due to the limited numbers. This represents one of the limitations of this study. Due to limited statistical power, it was not possible to correlate antibody levels before or after the third dose with severity of COVID-19 clinical presentation. As reported in a previously published article [24], only 64 patients (7.8%) out of all patients included in the cohort were hospitalized for COVID-19.

Our study identified diabetes and autoimmune diseases as significant predictors of reduced vaccine efficacy, consistent with the existing literature [14,15]. The role of hybrid immunity—combining natural infection and vaccination—in enhancing immune responses and improving outcomes in dialysis patients was also described [6,25,26]. Moreover, the exploration of variant-specific mono- or bivalent vaccines offered another avenue for boosting immunity, particularly against emerging SARS-CoV-2 variants. This approach has been crucial for dialysis patients, who remain at heightened risk for severe disease despite vaccination [4,21,27].

Our findings have significant implications for clinical practice and public health policy. Personalized vaccination strategies should be prioritized for dialysis patients, considering individual risk factors such as comorbidities and prior immune responses. Regular monitoring of antibody levels and timely administration of booster doses will be essential in optimizing protection in this group, to better understand the mechanisms of durability of immune responses post-booster and the potential benefits of hybrid immunity. Additionally, understanding the mechanisms underlying impaired vaccine responses in dialysis patients will be crucial in developing targeted interventions. This may involve exploring the role of uremia and other factors in modulating immune function and vaccine efficacy.

## 5. Conclusions

In conclusion, this study reinforces the critical role of the humoral response in preventing SARS-CoV-2 infections by SARS-CoV-2. The booster doses are fundamental in enhancing the immunity elicited by vaccination among fragile individuals including dialysis patients. By identifying factors associated with breakthrough infections, our research gives insights into designing targeted interventions aimed at reducing COVID-19 risk in this population.

## Figures and Tables

**Table 1:** Demographic and clinical characteristics of cases and controls and the total study cohort.

		Cases	Controls	*p*	Cohort Study [20]
Total		187	150		6555
No. of clinical centers		22	23		120
Age	mean (SD) median (min; max)	64 (15) 66 (22; 88)	68 (14) 70 (30; 96)	**0.023**	68 (14) 70 (18; 99)
Male sex	n (%)	119 (64)	91 (60)	0.573	4254 (65)
Heart disease	n (%)	86 (46)	49 (32)	**0.014**	2668 (41)
Diabetes	n (%)	58 (31)	28 (19)	**0.012**	1821 (28)
Arterial hypertension	n (%)	139 (74)	93 (62)	**0.013**	4381 (67)
Pulmonary diseases	n (%)	28 (15.0)	17 (11.3)	0.338	682 (10.4)
Autoimmune diseases	n (%)	17 (9)	4 (3)	**0.021**	352 (5)
Neoplasia	n (%)	29 (16)	37 (25)	**0.040**	1134 (17)
Cerebrovascular diseases	n (%)	22 (11.8)	10 (6.6)	0.135	677 (10)
Comorbidities	mean (SD) median (min; max)	3.0 (1.7) 3 (0; 8)	2.5 (1.8) 2 (0; 8)	**0.012**	2.8 (2.1) 3 (0; 8)
Comorbidities ≥ 2	n (%)	152 (81.3)	110 (72.9)	0.068	5921 (90)
Concurrent therapies *	mean (SD) median (min; max)	7.5 (3.4) 7 (0; 18)	6.8 (4.0) 7 (0; 16)	0.360	7.0 (3.7) 7 (0; 22)
Concurrent therapies * ≥ 5	n (%)	153 (81.8)	112 (74.2)	0.110	5136 (78.4)
Time (days) between second dose and T3	mean (SD) median (min; max)	142.9 (8.1) 142 (57; 167)	146.6 (12.8) 143 (116; 199)	**0.050**	
Time (days) between third dose and T4	mean (SD) median (min; max)	36.7 (20.5) 31 (9; 170)	73.0 (45.8) 72 (11; 196)	**<0.001**	

SD, standard deviation; * number of different active ingredients assumed in the six months preceding enrollment.

**Table 2:** Values of anti-S/RBD-IgG and neutralizing antibodies in cases and controls.

		Cases (187)	Controls (150)	*p* **
Anti-S/RBD-IgG ^§^ pre-third dose (T3)	mean (SD) median (min; max)	235.0 (686.5) 49.6 (0; 5939.5)	705.5 (1676.0) 102.1 (0.2; 10,173.5)	**0.0004**
Anti-S/RBD-IgG ^§^ post-third dose (T4)	mean (SD) median (min; max)	2291.7 (2761.6) 1363.9 (0; 15,000)	2454.8 (3174.2) 1142.6 (0.2; 15,000)	0.9527
log_2_ nAb * pre-third dose (T3)	mean (SD) median (min; max)	3.02 (1.56) 2.32 (2.32; 10.32)	3.79 (2.40) 2.32 (2.32; 10.32)	**0.0025**
log_2_ nAb * post-third dose (T4)	mean (SD) median (min; max)	6.23 (2.50) 7.32 (2.32; 10.32)	6.27 (2.60) 6.32 (2.32; 10.32)	0.9002

^§^ positive: ≥7.1 Binding Arbitrary Unit (BAU)/mL; * value expressed as log2 of the reciprocal of the dilution; ** *p* values correspond to the Mann–Whitney U test results for statistical significance between cases and controls for each antibody measurement at different time points.

**Table 3:** Unadjusted and adjusted OR of COVID-19 for levels of anti-S/RBD-IgG and log_2_ nAb and demographic and clinical characteristics.

			Anti-S/RBD-IgG	log_2_ nAb
		Unadjusted OR (95%CI)	Adjusted OR (95%CI)	Adjusted OR (95%CI)
Age (years)	<60	1	1	1
	≥60	0.69 (0.37–1.28)	0.87 (0.43–1.76)	0.85 (0.40–1.80)
Sex	Male	1	1	1
	Female	0.87 (0.46–1.65)	0.88 (0.43–1.78)	0.95 (0.45–2.00)
Anti-S/RBD-IgG pre-third dose (100 units) (T3)			0.97 (0.93–1.00)	
Anti-S/RBD-IgG post-third dose (100 units) (T4)			0.99 (0.93–1.00)	
log_2_ nAb pre-third dose *				0.81 (0.73–0.90)
log_2_ nAb post-third dose *				0.96 (0.83–1.11)
Diabetes	No	1	1	1
	Yes	2.00 (1.10–3.70)	1.82 (1.10–3.02)	1.97 (1.17–3.32)
Autoimmune diseases	No	1	1	1
	Yes	3.50 (1.40–9.00)	3.02 (1.15–7.91)	2.90 (1.19–7.08)
Neoplasia	No	1	1	1
	Yes	0.55 (0.33–0.93)	0.46 (0.21–1.01)	0.44 (0.19–1.00)
Time (days) between second dose and T3		0.96 (0.91–1.01)	0.97 (0.94–1.00)	0.97 (0.94–1.00)
Time (days) between third dose and T4		0.97 (0.95–0.99)	0.97 (0.95–0.99)	0.97 (0.95–0.99)

* The OR decrease for unit increase in log_2_ nAb, corresponding to a doubling of nAb.

## Data Availability

The data presented here do not include personal information about the patients. The datasets generated and/or analyzed during the current study are not publicly available but are available from the corresponding author upon reasonable request.

## Supplementary Materials

The following supporting information can be downloaded at: https://www.mdpi.com/article/10.3390/vaccines13090935/s1. Supplementary S1: Methods used for antibody assays. Reference [28] was cited in Supplementary S1. Supplementary S2: Membership of the COVIDVaxDia Study Group.
