# Supplementary material for: Humoral Response to the Third Dose of SARS-CoV-2 Vaccine Among Dialysis Patients: A Breakthrough Infection Case–Control Study
Source: Vaccines (Basel). 2025 Sep 1;13(9):935. doi: 10.3390/vaccines13090935 (PMC12474067; doi:10.3390/vaccines13090935)
Supplement: Supplementary file 1 [file vaccines-13-00935-s001.zip › vaccines-3760050-supplementary.pdf]

Supplementary S1

Serological tests for SARS-CoV-2

Two commercial chemiluminescence microparticle antibody assays, the SARS-CoV-2–specific anti-N-IgG and the anti-S/RBD-IgG tests (ARCHITECT SARS-CoV-2 IgG and ARCHITECT SARS-CoV-2 IgG II Quantitative; Abbott Laboratories, Wiesbaden, Germany, respectively), were performed on the ARCHITECT i2000sr (Abbott Diagnostics, Chicago, IL) according to the manufacturer's instruction. Anti-N-IgG and the anti-S/RBD-IgG are considered positive with index values >1.4 and binding antibody units (BAU)/mL ≥7.1 are considered positive for, respectively. A microneutralization assay (MNA) was performed, as previously described, to determine the SARS-CoV-2 neutralizing antibodies (nAb) titers, using Wuhan-D614G strain (GISAID accession ID EPI_ISL_ 568579; Ref-SKU: 008V-04005) as the challenging virus [1]. Briefly, serum samples were heat-inactivated at 56°C for 30 minutes and titrated in duplicate in 7 two-fold serial dilutions (starting dilution 1:10). Equal volumes (50 μL) of serum and medium containing 100 median tissue culture infective dose50 (TCID50) SARS-CoV-2 were mixed and incubated at 37°C for 30 minutes. Serum–virus mixtures were then added to sub-confluent Vero E6 cell monolayers and incubated at 37°C with 5% CO2. After 48 hours, microplates were observed for the presence of cytopathic effect using a light microscope. To standardize inter-assay procedures, positive control samples that showed high (1:160) and low (1:40) neutralizing activity were included in each assay session. Serum from the National Institute for Biological Standards and Control (United Kingdom; NIBSC) with known neutralization titer (research reagent for anti–SARS-CoV-2 Ab NIBSC code 20/130) was used as the reference in MNA. The neutralization titer was assigned as the highest serum dilution inhibiting at least 90% of the cytopathic effect (NT90), with a positivity threshold set at 1:10.

1. Matusali, G.; Colavita, F.; Lapa, D.; Meschi, S.; Bordi, L.; Piselli, P.; Gagliardini, R.; Corpolongo, A.; Nicastri, E.; Antinori, A.; et al. SARS-CoV-2 Serum Neutralization Assay: A Traditional Tool for a Brand-New Virus. Viruses 2021, 13, 655, doi:10.3390/v13040655.

Supplementary S2

The COVIDVaxDia Study Group comprises the following individuals:

Francesca Menniti-Ippolito, Roberto Da Cas, Flavia Chiarotti, Massimo Fabiani, Giuseppe Traversa (Italian National Institute of Health, Rome, Italy), Piergiorgio Messa (Fondazione IRCCS Ca' Granda Ospedale Maggiore Policlinico di Milano), Alfonso Mele (Epidemiologist, retired from Italian National Institute of Health, Rome, Italy), Salvatore De Masi (Clinical Trial Centre, Careggi Hospital, Florence, Italy), Francesca Colavita (Laboratory of Virology, National Institute for Infectious Diseases Lazzaro Spallanzani, IRCCS, Rome, Italy), Concetta Castilletti (Department of Infectious, Tropical Diseases and Microbiology, IRCCS Sacro Cuore Don Calabria Hospital, Negrar di Valpolicella, Verona, Italy), Carmine Zoccali (Associazione Ipertensione, Nefrologia e Trapianto Renale (IPNET) c/o Nefrologia, Reggio Calabria, Italy), Aldo Pietro Maggioni, Andrea Lorimer, Martina Ceseri, Ester Baldini, Francesca Bianchini, Laura Sarti (ANMCO Research Center, Heart Care Foundation Onlus, Florence, Italy), Giovanni Baglio (Italian National Agency for Regional Healthcare Services, Rome, Italy), Andrea Mariano (National Institute for Infectious Diseases Lazzaro Spallanzani, IRCCS, Rome, Italy), and Eva Alessi (Italian Medicine Agency, Rome, Italy).

The following dialysis clinical centers took part:

◎ SS Annunziata-Chieti: Lorenzo Di Liberato.

◎ Ospedale Civile "G. Mazzini"-Teramo: Luigi Amoroso, Nicola Spetrino, Milva Di Giovanni.

◎ Ospedale Ferrari-Castrovillari (CS): Carlo Sapio, Irma Figlia, Roberto Pititto.

◎ Azienda Ospedaliera di Cosenza: Teresa Papalia, Rosita Greco, Francesca Leone.

◎ Giovanni Paolo II-Lamezia Terme: Francesco Maria D'agostino, Maria Angela Campolo, Maria Rosa La Gamba.

◎ Grande Ospedale Metropolitano-Reggio Calabria: Francesca Mallamaci, Vincenzo Panuccio,

Giovanna Parlongo.

◎ G. Jazzolino-Vibo Valentia: Giuseppe Natale, Domenico Tramontana, Ivania Figliano.

◎ Nephrocare-Angri: Santo Vitiello, Annamaria Frangiosa.

◎ AORN Sant'Anna e San Sebastiano-Caserta: Domenico Caserta, Pasqualina Acconcia, Nunzia

Paudice.

◎ Centro Medico Metelliano-Cava dei Tirreni (SA): Alfonso De Maio, Salvatore Cascone,

Marilina Siani.

◎ NephroCare S.p.A.-Cercola (NA): Mario Cioffi, Nataliya Romanyuk, Franca Pagnano.

◎ NephroCare S.p.A.-Fisciano (NA): Antonio De Donato.

◎ Ambulatorio di Emodialisi Nefrocenter, Frattamaggiore (NA): Ersilia Satta.

◎ AUO Policlinico Vanvitelli-Napoli: Alessandra Perna, Francesco Trepiccione, Alessandro

Cerrone.

◎ Nephrocare S.p.A.-Napoli: Gianluca Garofalo.

◎ Nephrocare-Napoli: Giancarlo Marinelli.

◎ Nephrocare S.p.A.-Napoli: Roberta D'amato.

◎ Nephrocare-Napoli: Enrico De Felice, Alfredo Vacca.

◎ P.O. Piedimonte Matese-Caserta: Salvatore Coppola, Vincenzo Cuomo.

◎ Nephrocare S.P.A.-Pozzuoli (NA): Maria Palma Iavarone.

◎ NephroCare S.P.A.-Quarto (NA): Annalisa Ciotola.

◎ NephroCare S.P.A.-Roccadaspide (SA): Vincenzo Puglia, Pio Granato.

◎ Nephrocare Emodial-Salerno: Carla Lamberti, Giorgio Capasso.

◎ NephroCare S.p.A.-Salerno: Domenico Bonanno, Marco Ventre, Veronica Amendola.

◎ Nephrocare-Torre del Greco (NA): Fabio Cappabianca.

◎ IRCCS-Azienda Ospedaliero-Universitaria Policlinico S. Orsola-Bologna: Elena Mancini,

Davide Ricci, Daniela Cecilia Cannarile, Roberta Benevento, Bianca Perciaccante.

◎ AOU Sant'Anna-Cona (FE): Alda Storari, Yuri Battaglia, Giovanni Piva.

◎ Ospedali di Forlì e Cesena-Forlì: Giovanni Mosconi, Alessandra Spazzoli, Paolo Ferdinando

Bruno, Katia Ambri, Barbara Veterani, Sara Signorotti.

◎ S. Maria della Scaletta-Imola: Marcora Mandreoli, Renato Mario Rapanà, Maria Teresa

Benedetto, Laura Patregnani, Maddalena Zambelli.

◎ Azienda Ospedaliero-Universitaria di Modena: Gaetano Alfano.

◎ Azienda USL–IRCCS di Reggio Emilia, Nephrology and Dialysis Unit: Silvia Mattei, Francesco Iannuzzella, Elena Pelizzaro, Luca Camparini, Giuseppe Battaglino, Mariacristina Gregorini.

◎ San Daniele-Tolmezzo (UD): Dino Romanini, Antonio Irlando, Lojze Celik, Giuseppa Natale.

◎ Centro Dialisi Ambulatoriale Geramed Srl-Fiano Romano (RM): Maria Cristina Torre, Ilaria

Umbro.

◎ Casa di cura Nuova Itor-Roma: Nicola Pirozzi, Loredana Fazzari, Lucia Pantano.

◎ Ospedale Sandro Pertini-Roma: Antonio Paone, Marco Galliani, Veronica Baglio, Eleonora

Moscaritolo, Sabrina Fierimonte, Maria De Cristofaro, Elena Nebuloso.

◎ Azienda Ospedaliero-Universitaria Sant'Andrea-Roma: Paolo Menè, Francesca Romana

Festuccia, Giulia Talarico, Claudia Fofi, Maria Elena Bracaccia.

◎ SS Trinità-Sora (FR): Ernesto Anselmo Cioffi, Fabio Mazza.

◎ San Giovanni Evangelista-Tivoli (RM): Pasquale Polito, David Micarelli, Roberto Addesse,

Lida Tartaglione.

◎ Belcolle-Viterbo: Sandro Feriozzi, Francesca Romana Della Rovere, Natalia Chipilova, Micol

Manzuoli.

◎ Ospedali Villa Scassi (Genova) e La Colletta (Arenzano, GE): Paolo Sacco, Francesca Ansaldo,

Chiara Bottaro, Sonia Marre.

◎ IRCCS Ospedale Policlinico San Martino-Genova: Francesca Viazzi, Valeria Falqui, Novella

Conti, Angelica Parodi, Valentina Zanetti, Francesca Cappadona, Andrea Speciale.

◎ Ospedale S. Paolo-Savona: Giancarlo Mancuso, Monica Repetto, Emanuela Chiara Vigo, Anna

Maria Murgia.

◎ Papa Giovanni XXIII-Bergamo: Piero Ruggenenti, Patrizia Ondei, Carmela Giuseppina Condemi, Silvia Bernardi.

◎ ASST Spedali Civili di Brescia: Francesco Scolari, Paola Gaggia, Federico Alberici, Chiara

Manenti, Brunella Valzorio, Corrado Camerini, Agnese Gallico, Michela Tonoli, Federico Daffara, Roberto Zubani, Simona Guerini, Mattia Zappa, Nicole Zambetti.

◎ San Pellegrino s.r.l.-Castiglione delle Stiviere (MN): Alessandra Dalla Gassa, Paola Baldan,

Luca Fraizzoli.

◎ ASST Melegnano e della Martesana P.O. "Uboldo" di Cernusco sul Naviglio (Mi): Sergio

Bisegna, Nicola Palmieri, Marco Petrilli, Giusy Mandanici, Francesca Serena Stefani.

◎ ASST Crema (CR): Valeria Ogliari, Cristina Tantardini.

◎ ASST-Cremona: Fabio Malberti, Paola Pecchini.

◎ Ospedale A. Manzoni, ASST di Lecco: Vincenzo La Milia.

◎ ASST OVEST Milanese Ospedali di Legnano e di Magenta (MI): Carlo Maria Guastoni, Annalisa Neri, Marina Cornacchiari.

◎ ASST di Lodi: Marco Farina, Francesco Barbisoni.

◎ ASST Santi Paolo e Carlo-Milano: Mario Cozzolino, Matthias Cassia, Michela Frittoli, Lorenza

Magagnoli, Rossella De Leonardis, Roberta Casazza.

◎ Fondazione IRCCS Ca' Granda Ospedale Maggiore Policlinico di Milano: Piergiorgio Messa,

Simone Vettoretti, Emanuele Grimaldi, Matteo Abinti, Francesca Maria Ida Carminati, Silvia

Giuliani, Matteo Benedetti, Nicholas Walter Delfrate, Elisa Colombo, Angela Cervesato.

◎ ASST-GOM Niguarda-Milano: Enrico Eugenio Minetti, Alberto Montoli, Chiara Brunati,

Valeria Li Bergolis.

◎ Presidio Fatebenefratelli-Milano: Maurizio Gallieni, Laura Cosmai, Maria Antonietta Orani,

Cristina De Salvo.

◎ ASST Fatebenefratelli, PO Sacco-Milano: Maurizio Gallieni, Cristina Airaghi, Monique Buskermolen.

◎ IRCCS Ospedale San Raffaele-Milano: Giuseppe Vezzoli, Giorgio Slaviero, Chiara Lanzani.

◎ Fondazione IRCCS San Gerardo dei Tintori–Monza: Federico Pieruzzi, Gina Contaldo, Barbara Trezzi.

◎ Fondazione IRCCS Policlinico San Matteo-Pavia: Teresa Rampino, Fabrizio Grosjean, Paola

Borille.

◎ ICS Maugeri SpA SB-Pavia: Ciro Esposito, Giuseppe Sileno, Marta Arazzi.

◎ ASST Lariana Osp. Sant'Anna-Como, San Fermo della Battaglia (CO): Gianvincenzo Melfa,

Mariagiulia Tettamanti, Anna Tosetti, Marco D'Amico, Maria Giulia Magatti, Silvia Peiti.

◎ Nephrocare SPA c/o ASST Bergamo Est-Seriate: Luciano Pedrini, Annalisa Feliciani, Elena

Pezzini.

◎ IRCCS Multimedica-Sesto San Giovanni (MI): Silvio Volmer Bertoli, Daniele Ciurlino, Silvia

Tedoldi, Vania Prettico, Giulia Maria Magni.

◎ Vigevano (PV): Antonietta Gazo, Maurizio Nai, Silvia Muciaccia.

◎ Ospedale di Vimercate (MB): Graziana Battini, Lino Merlino, Paola Casanova.

◎ IRCCS INRCA-Ancona: Federica Lenci, Maddalena Ricci, Roberta Galeazzi, Anna Rita

Bonfigli, Fabrizia Lattanzio.

◎ Ospedali Riuniti Ancona: Andrea Ranghino, Mauro Valente, Carolina Finale.

◎ Civile "Egles Profili"-Fabriano (AN): Eleonora Guerrini, Marta Canonici.

◎ Carlo Urbani-Jesi (AN): Stefano Santarelli, Rosa Maria Agostinelli.

◎ A.S.T. Pesaro Urbino-Ospedali S. Salvatore Pesaro e S. Croce Fano-Pesaro (PU): Marina Di

Luca, Mauro Martello, Cristina Silvestri, Veronica Bertuzzi, Assunta Cardillo, Valentina Nastasi,

Sara Belcastro.

◎ AO "SS. Antonio e Biagio e Cesare Arrigo"-Alessandria: Marco Manganaro, Emanuele Luigi

Parodi, Valentina Vaccaro, Antonella Giolito.

◎ Presidio Ospedaliero S.S. Trinità di Borgomanero (NO): Stefano Cusinato, Michele Battista,

Elena Ragazzoni, Paola Marcella Carpani.

◎ Ospedale Maggiore, ASLTO 5-Chieri (TO): Mario Salomone, Emanuele Stramignoni.

◎ ASL TO4 (TO) sede Cirié (TO): Silvana Savoldi, Guido Martina, Marica Magnetti; sede Ivrea

(TO) Chiara Deagostini; sede Chivasso (TO), Stefania Bussolino, Federica Ventrella, Sonia Santi,

Giacinta Vigilante. ASLCN1-Cuneo: Marita Marengo, Daniela Falconi, Ilaria Serra.

◎ AO S. Croce e Carle-Cuneo: Luca Besso, Davide Diena.

◎ AOU Maggiore Della Carita'-Novara: Doriana Chiarinotti, Paola David, Luciana Gravellone.

◎ Azienda Ospedaliera Ordine Mauriziano di Torino: Corrado Vitale, Silvia Berutti, Silvia Ganci.

◎ Martini-Torino: Giulio Cesano, Federica Neve Vigotti.

◎ ASL VCO: Maurizio Borzumati, Patrizia Vio, Stefania Gioira.

◎ SC Nefrologia e Dialisi, ASL Vercelli: Simonetta Ottone, Oliviero Filiberti.

◎ Ente Ecclesiastico Ospedale F. Miulli-Acquaviva delle Fonti (BA): Carlo Lomonte, Vincenzo

Montinaro, Vito Pepe, Elisabetta Manno.

◎ Bonomo-Andria (BAT): Salvatore Di Paolo, Luigi Natalicchio, Carmela Gallo, Francesca

Pansini, Rossella Varvara.

◎ Di Venere ASL Bari: Filomena D'Elia, Nicola Coviello, Concetta Prisciandaro, Lucia Vernò.

◎ New Dial-Bari: Fernanda Misceo, Pierfelice Zazzera, Antonella Matrella.

◎ AOU Consorziale Policlinico di Bari: Loreto Gesualdo, Anna Maria Di Palma, Virginia Pronzo,

Federica Cassone.

◎ "Dimiccoli"-Barletta: Salvatore Di Paolo, Tiziana Piccolo, Dora Porcelluzzi, Antonella Di

Franco, Mario Giannetto, Villani Chiara.

◎ "A. Perrino"-Brindisi: Luigi Vernaglione.

◎ P.O "Tatarella"-Cerignola e P.O "San Camillo De Lellis "-Manfredonia (FG): Angelo Specchio, Laura Stoico.

◎ Dipartimento di Scienze mediche e Chirurgiche-Università di Foggia: Giovanni Stallone,

Vincenzo Losappio, Barbara Infante.

◎ Valle d'Itria-Martina Franca (TA): Anna Lisa Marangi, Angela Rodio, Christian D'Altri,

Francesca Salvatore, Maria Antonietta Perniola, Antonella Netti.

◎ Casa Sollievo della Sofferenza IRCCS-San Giovanni Rotondo (FG): Filippo Aucella, Gaetano

Ferrara, Anna Rachele Nardella.

◎ ASL Taranto, P.O. Centrale S.S. Annunziata di Taranto: Luigi Francesco Pio Morrone, Maria

Grazia Corallo, Giuseppina D'Ettorre.

◎ Pia Fondazione Cardinal Panico-Tricase (LE): Francesco Caccetta, Davide Gianfreda.

◎ Dipartimento di Scienze Mediche e Sanità Pubblica Università degli Studi di Cagliari -

A.R.N.A.S. Brotzu Cagliari: Antonello Pani, Gianfranca Cabiddu.

◎ Nephrocare Spa-Casoria (NA): Maria Maddalena Romano, Ciro Adamo.

◎ P.O. SS Marta e Venera-Acireale (CT): Giovanni Giorgio Battaglia, Barbara Pocorobba,

Emanuela Prencipe, Margherita Saraceno, Luana Scuderi.

◎ PO "G. di Maria" Avola, (SR): Salvatore Randone, Francesca Bruno, Rossella Rita Marchese.

◎ Az. Ospedaliera per l'Emergenza "Cannizzaro"-Catania: Antonio Granata, Daniela Puliatti.

◎ Azienda Ospedaliero-Universitaria Policlinico "G. Rodolico San Marco"-Catania: Carmelita

Marcantoni, Luca Zanoli, Ambra D'Anca.

◎ Aou G Martino-Messina: Domenico Santoro, Guido Gembillo, Alfio Edoardo Giuffrida,

Vincenzo Labbozzetta.

◎ ARNAS Civico Di Cristina-Palermo: Angelo Tralongo, Gioacchino Li Cavoli, Carmela

Zagarrigo.

◎ Ospedale di Arezzo, Azienda USL Toscana SudEst-Arezzo: Paolo Conti, Sofia Giovannini,

Silvia Farsetti.

◎ Santa Maria Annunziata-Bagno a Ripoli (FI): Pietro Claudio Giovanni Dattolo, Aris Tsalouchos, Stefano Michelassi.

◎ Istituto Fiorentino di Cura e Assistenza-IF.C.A.-Firenze: Marco Gallo, Irene Bandor.

◎ Ospedale Apuane Azienda USL Nordovest Toscana-Massa: Aldo Casani, Nadia Sami, Daniela

Riccomi.

◎ U.O.S.D. Nefrologia e Dialisi S.O. S. Andrea Massa Marittima A.U.S.L. Toscana sudest: Lorena

Traversari.

◎ San Maurizio-Bolzano: Maria Grazia Tabbí, Maria Luisa Bonincontro, Vera Bonell.

◎ Azienda Provinciale per i Servizi Sanitari (APSS), Trento: Giuliano Brunori, Diana Zarantonello, Alessandro Laudon, Alessia Dalpiaz.

◎ Media Valle del Tevere-Assisi-Castiglione del Lago-Todi (PG): Antonio Selvi, Stefania

Santoni, Davide Massimiani, Davide Rossi.

◎ Azienda Ospedale-Università Padova: Lorenzo Calò, Barbara Rossi, Giuseppe Scaparrotta.

◎ UOC Nefrologia e Dialisi, AULSS 5 Polesana, Ospedale SM della Misericordia-Rovigo: Fulvio

Fiorini, Valentina Iacono.

◎ San Bonifacio (Verona): Carlo Rugiu, Linda Gammaro, Monica Slaviero, Vincenzo Cosentini.

◎ Ospedale Ca' Foncello-Treviso: Maurizio Nordio, Riccarda Maria Puggia, Adriana Caberlotto.

◎ SS. Giovanni e Paolo-Venezia: Flavio Scanferla, Vincenzo Casoria, Federica Gramegna.

◎ Ospedale di Dolo e Mirano-Venezia: Gina Meneghel, Ilenia Filippi, Anna Giacomini.

◎ San Bortolo-Vicenza: Claudio Ronco, Anna Giuliani, Sabrina Milan Manani, Monica Zanella.
